# Checking Perspiration

**Published:** 1880-06

**Authors:** 


### CLERICAL RECREATIONS.

To no class of persons does this nation owe more of its stability and greatness than to its clergy; their learning, their talent, their piety, their love of liberty and the right, their resistance against oppression and the wrong, are the glory of any people, and more essential to national advancement than a million times their number of bar room politicians and quibbling lawyers. But with the talent and capabilities, which, if exerted in other directions, would place them at the head in the counting-room and on 'change, they do not, on an average, get the annual pay of a New York drayman. Such being the case—and shame is it to the intelligence and piety of this land that it is so—we have no right to direct them as to the expenditure of their time. But willing to do them a service, to suggest somewhat that may add to their health and usefulness, we propose the following as a very profitable method of recreating themselves during the summer:

Let them travel together, two and two, on horseback, through the destitute and mountainous parts of the country, preaching in the forenoon, and at night of each day; in the forenoon, at some country church or tavern, or cross roads, or post-office; and at night, in some town or village.

There is no more delightfully healthful form of exercise than that of moderate horseback travel, day after day, some eighteen miles between breakfast and dinner, and some twelve miles between dinner and supper. The change of scene, of employment, of air, of food, of mode of preparation, the relaxation from severe study to that of a moderate and unlaborious sort, the freshness which will invest old ideas, and old skeletons and old sermons, when connected with the consciousness that they are perfectly new to the auditory, the pleasurable feeling which pervades the heart in the reflection that the seed of the word is thus sown to many who else might not have had it scattered to them again, perhaps in a life-time, with the assurance that it must take root in some hearts, we repeat it, all these things together, when a minister *has a mind to the work*, when it is his *meat and drink* to be thus employed, will work such a change in the physical conviction of a man as will enable him to return to the people of his charge with a store of health, with a vigor of mind, with a warmth of heart and elevation of spirit, of which those clergymen have no conception whose recreations are to feed and lounge on the sea shore, or at the Spa. Let each congregation that feels their minister ought to have a holiday during the heats of summer, provide him with a hundred or two dollars extra, and say to him, *as ye go*, PREACH!

### CARE OF THE FEET.

One evening in Boston, just as *Washington Alston*, the Painter, was approaching the door of a dwelling, where a splendid party had assembled, he suddenly stopped short and said to his friend,

"I cannot go in."

Nonsense! why not?

"I have a hole in one of my stockings."

Pshaw, man, nobody knows it.

"But I do," said the celebrated Artist.

